# Editorial: Mental, sensory, physical and life style parameters related to cognitive decline in aging

**DOI:** 10.3389/fnagi.2026.1803069

**Published:** 2026-02-27

**Authors:** Panagiotis Alexopoulos, Chinedu T. Udeh-Momoh, Maria Basta

**Affiliations:** 1 Mental Health Services, Faculty of Medicine, School of Health Sciences, University of Patras, Patras, Greece; 2 Global Brain Health Institute, School of Medicine, Trinity College Dublin, The University of Dublin, Dublin, Ireland; 3 Department of Psychiatry and Psychotherapy, Klinikum Rechts der Isar, Technical University of Munich, Munich, Germany; 4 Patras Dementia Day Care Centre, Patras, Greece; 5 School of Public Health Sciences, Wake Forest University School of Medicine, Winston Salem, NC, United States; 6 Brain and Mind Institute, Aga Khan University, Nairobi, Kenya; 7 Division of Clinical Geriatrics, Centre for Alzheimer Research, Karolinska Institutet, Solna, Sweden; 8 Sheffield Institute for Translational Neuroscience (SITraN), University of Sheffield, Sheffield, United Kingdom; 9 Department of Psychiatry, University Hospital of Heraklion, Medical School, University of Crete, Heraklion, Greece; 10 Day Care Center for Alzheimer's Disease PAGNH “Nefeli”, University Hospital of Heraklion, Crete, Greece

**Keywords:** brain health, cognitive and mental health in aging, collaborative interventions, interdisciplinarity, prevention

Brain health is an emerging, overarching and complex concept including prevention of cognitive decline and neurocognitive disorder (Chen et al., 2021; World Health Organization, 2022). It is significantly affected by all modifiable risk factors for dementia, which account for approximately 45% of all dementia cases across the globe (World Health Organization, 2022; Alexopoulos et al., 2024; Livingston et al., 2024). This Research Topic sheds light on the multifactorial nature of cognitive decline in aging and on how sensory function, physical activity and performance, cardiometabolic health, mental health, and multimorbidity interact to influence cognitive trajectories in aging.

Age-related sensory loss is a strong and possibly causal predictor of cognitive decline, and early detection—even via self-report—is feasible and valuable. Auditory loss has a considerable impact on cognition as shown by cross-sectional data deriving from the AD-HEARING study (Belz et al.), as well as from genomic and epidemiological investigations in the Genome-wide association studies database and in communities in China, respectively (Xu et al.). In addition, visual dysfunction is likewise implicated in cognitive impairment and impairment of daily living activities in people with MCI (Zhou, Yang et al.). Of note, in older individuals without cognitive impairment, subjective visual impairment complaints are positively related to visual performance, whereas in those carrying the APOE e4 allele such complaints pertain to attention, processing speed and executive function (Dubois et al.). Thus, it becomes evident that assessment of sensory function embodies a component of the diagnostic workup of cognitive decline in aging (Dubois et al.). Pragmatic and effective tools are urgently needed for facilitating the early detection of sensory loss. An ultra-brief screening tool based on demographic data, one item of the Hearing Handicap Inventory for the Elderly (HHIE) and one of the HHIE for the communication partner is here presented. It may help the greater integration of hearing assessment and can complement the cognitive function assessment with simple and quickly administered tests like the ten-words recall test (Ren et al.), in community and primary healthcare settings where the preponderance of older people in fact seek care (Aggeletaki et al., 2024) (Alexopoulos, Demertzis et al.). Of note, Lei Kei et al. unveiled that the combined effect of sensory loss and low physical performance on cognitive function is additive and this effect is mitigated by participation in social activities such as interacting with friends, playing chess or cards and being involved in voluntary or charity work (Lei et al.).

Mounting evidence points to the protective influence of physical activity, muscle strength, and overall physical performance on cognitive function in aging. Recreational physical activity of moderate to high intensity is associated with better performance on semantic fluency, executive function and processing speed and this relationship is partly mediated by depressive symptoms (Zhang, Tian et al.). Moreover, in another article of this Research Topic, grip strength and performance on the 30-s sit-to-stand test, assessing lower body strength, balance and endurance, were shown to relate with processing speed, sustained attention and general cognition (Xin et al.) (Benedict et al., 2017; Hagenaars et al., 2017). Interestingly, EEG markers were shown to reflect both muscle strength and information processing. They provide valuable insights into their potential utility in deciphering the neuropathophysiological interplay between muscle strength and cognitive function. A systematic review and network meta-analysis provide evidence for the optimal dose and type of exercise to improve cognitive function in people with mild neurocognitive disorder, commonly embodying a pre-dementia stage in people with neurodegenerative disorders (Guo et al., 2013; Yu et al.). In particular, 12–24 week-, multi-component exercise sessions with 60–85% of maximum heart rate which last 30 min and take place 3–4 times per week are beneficial for global cognition, while sessions lasting 30–61 min, 3–4 times per week for 25 weeks or longer, show greater effectiveness in enhancing executive function. Nonetheless, no consensus can be reached yet regarding the optimal dose of physical exercise, since in another study, even though individuals with a longer history of exercise were less likely to experience cognitive decline, prolonged exercise duration was found to increase the risk of cognitive decline, since it may overload the body (Tong et al.).

Physical health and healthy dietary habits are crucial for maintaining cognitive function in aging. For instance, calf circumference, a screening method for sarcopenia (Piodena-Aportadera et al., 2022), and bone mineral density, a marker of osteoporosis, are both associated with global cognition in women. Notably, in females correlations between both calf circumference and bone mineral density and global cognition and several neuropsychological scores, i.e., executive function, memory, attention, and brain speed were detected (Nie et al.; Chen et al.). Moreover, previous reports highlight the role of insulin in amyloid β peptide clearance, tau phosphorylation, and the modulation of vascular function, which pertain to cognitive decline and progression to dementia (Kellar and Craft, 2020). In line with that, triglyceride-glucose index, a marker of insulin resistance, was found to be associated with impairment in both basic and complex activities of daily living (Wei et al.). Furthermore, hypertension, cardiovascular disease, unhealthy diet, smoking or decreased cerebral blood flow during hemodialysis are contributing factors to cognitive decline (Tong et al.; Guo et al.; Hu et al.). Furthermore, higher values of body roundness index, reflecting fat distribution, are linked to worse performance on several neuropsychological scales, and this negative association is stronger in males (Zhang, Ning et al.). It is noteworthy that the higher the total number of morbidities, the higher the risk for cognitive decline and disturbances in activities of daily living (Zhou, Qin et al.; Lai et al.).

Depressive symptoms and sleep disturbances have emerged as consistent risk factors for cognitive decline in aging, while they even serve as mediators in the relationship between physical/medical factors and cognitive decline. Depression in old adults, which often manifests with physical discomfort and somatic symptoms (Guo and He), has increasingly attracted attention from researchers (Du et al.). Besides its relation to intrinsic capacity, which refers to the set of all the physical and mental capabilities (Liu, Li et al.), depression partially mediates the relationship between chronic diseases, such as poor cardiovascular health, and cognitive impairment (Hu et al.; Lai et al.). Even though late-onset depression is closely linked to vascular and degenerative brain changes and chronic diseases (Linnemann and Lang, 2020), social participation patterns are related to the severity of depressive symptoms particularly among frail older individuals (Liu, Zhou et al.). In addition, sleep disturbances are increasingly considered to relate with cognitive impairment. In a cross-sectional study including a total of 3,106 individuals, subjective short sleep duration, combined with poor sleep quality and sleep medication were associated with cognitive dysfunction (Jiang et al.).

It is evident that both cognitive decline and brain health maintenance are contingent on a complex interplay between a plethora of factors that should be considered to promote healthy cognitive and mental aging. Multicomponent interventions have already taken place or are underway in various countries aiming at reducing the risk of dementia through interventions targeting modifiable risk factors. The Greek Interventional Geriatric Initiative to Prevent Cognitive Impairment and Disability in individuals with subjective cognitive decline is such a study (Alexopoulos, Felemegkas et al.). Interventions are tailored to the individual needs of each beneficiary, implementing a precision-medicine approach and benefits from tele-health tools (Alexopoulos, Felemegkas et al.; Liu, Liu et al.).

Collectively, the Research Topic Cognitive Aging, Sensory and Physical Function, Mental Health, and Related Risk Factors contributes to a robust, multidimensional understanding of cognitive decline in older adults. It highlights the interconnected impact of sensory function, physical performance, cardiometabolic health, depression, social engagement, and lifestyle factors on cognitive function. While challenges remain—particularly in early detection, mechanistic clarity, and intervention scalability-, these findings can inform the development of pragmatic, integrated, personalized, community brain health services that are adjusted to local contexts, meet local needs, and fuel future research endeavors.

## References

[B1] Aggeletaki E. Stamos V. Konidari E. Efkarpidis A. Petrou A. Savvopoulou K. . (2024). Telehealth memory clinics in primary healthcare: real-world experiences from low-resource settings in Greece. Front. Dement. 3:1477242. doi: 10.3389/frdem.2024.1477242 39665073 PMC11631602

[B2] Alexopoulos P. Leroi I. Kinchin I. Canty A. J. Dasgupta J. Furlano J. A. . (2024). Relevance and premises of values-based practice for decision making in brain health. Brain Sci. 14:718. doi: 10.3390/brainsci14070718 39061458 PMC11274584

[B3] Benedict R. H. B. Deluca J. Phillips G. LaRocca N. Hudson L. D. Rudick R. (2017). Validity of the Symbol Digit Modalities Test as a cognition performance outcome measure for multiple sclerosis. Mult. Scler. 23:721. doi: 10.1177/1352458517690821 28206827 PMC5405816

[B4] Chen Y. Demnitz N. Yamamoto S. Yaffe K. Lawlor B. Leroi I. (2021). Defining brain health: a concept analysis. Int. J. Geriatr. Psychiatry 37. doi: 10.1002/gps.5564 34131954

[B5] Guo L. H. Alexopoulos P. Eisele T. Wagenpfeil S. Kurz A. Perneczky R. (2013). The National Institute on Aging-Alzheimer's Association research criteria for mild cognitive impairment due to Alzheimer's disease: predicting the outcome. Eur. Arch. Psychiatry Clin. Neurosci. 263, 325–333. doi: 10.1007/s00406-012-0349-0 22932720

[B6] Hagenaars S. P. Cox S. R. Hill W. D. Davies G. Liewald D. C. M. Harris S. E. . (2017). Genetic contributions to Trail Making Test performance in UK Biobank. Mol. Psychiatry 23, 1575–1583. doi: 10.1101/103119 28924184

[B7] Kellar D. Craft S. (2020). Brain insulin resistance in Alzheimer's disease and related disorders: mechanisms and therapeutic approaches. Lancet Neurol. 19, 758–766. doi: 10.1016/S1474-4422(20)30231-3 32730766 PMC9661919

[B8] Linnemann C. Lang U. E. (2020). Pathways connecting late-life depression and dementia. Front. Pharmacol. 11:520821. doi: 10.3389/fphar.2020.00279 32231570 PMC7083108

[B9] Livingston G. Huntley J. Liu K. Y. Costafreda S. G. Selbæk G. Alladi S. . (2024). Dementia prevention, intervention, and care: 2024 report of the Lancet standing Commission. Lancet 404, 572–628. doi: 10.1016/S0140-6736(24)01296-0 39096926

[B10] Piodena-Aportadera M. R. B. Lau S. Chew J. Lim J. P. Ismail N. H. Ding Y. Y. . (2022). Calf circumference measurement protocols for sarcopenia screening: differences in agreement, convergent validity and diagnostic performance. Ann. Geriatr. Med. Res. 26:215. doi: 10.4235/agmr.22.0057 36031936 PMC9535367

[B11] World Health Organization R. R.. (2022). Optimizing Brain Health Across the Life Course, 1–99. Available online at: https://www.who.int/publications/i/item/9789240054561 (Accessed December 18, 2023).

